# The influence of the corporate social responsibility disclosures on consumer brand attitudes under the impact of COVID-19

**DOI:** 10.1186/s11782-020-00096-0

**Published:** 2020-12-28

**Authors:** Fengjun Liu, Lu Meng, Yijun Zhao, Shen Duan

**Affiliations:** Business School, Renmin University of China, Beijing, 100872 China

**Keywords:** 2019 novel coronavirus disease (COVID-19), Small- and medium-sized enterprises (SMEs), Internal corporate social responsibility (ICSR), Enterprise social responsibility disclosures, Consumer brand attitudes

## Abstract

This study focuses on the use of we-media by small- and medium-sized enterprises (SMEs) to disclose internal corporate social responsibility (ICSR) under the impact of the 2019 novel coronavirus disease (COVID-19). Study 1 interprets the catalyst effect of COVID-19 on the externalization of SMEs’ ICSR. The fuzzy grading evaluation method is initially verified. Under the impact of COVID-19, SMEs fulfilling their ICSR can enhance consumer brand attitudes. Study 2 uses a structural equation model and empirical analysis of 946 effective samples and finds that consumers perceive the self-sacrifice of corporations during the coronavirus disease period. SMEs can fulfill their ICSR to enhance the internal explanation mechanism of consumer brand attitudes and the moderating role of enterprise losses.

## Introduction

The 2019 novel coronavirus disease (COVID-19) has caused a sudden outbreak of pneumonia. The World Health Organization has declared a public health emergency. The disease has posed great risks and challenges to the world’s economic and social development (Ding et al. 2020). Based on past experience of major disasters, enterprises take the initiative to carry out their social responsibilities, quickly resolve their difficulties as it is directly related to enterprises’ future development in the post-pandemic era. These actions will have a profound impact on the brand image of enterprises. Enterprises’ active disclosure of their corporate social responsibility (CSR) activities through social media can affect advertising and improve enterprise image and performance (Gong et al. 2018). However, this development strategy does not apply to small- and medium-sized enterprises (SMEs) (Guo et al. 2020; Heidi et al.^ 2011^). The practice of social responsibility needs to be linked to enterprises’ development and operation to ensure that SMEs have the power and ability to create social value in the new epidemic situation continuously. Therefore, all SMEs should fulfill their social responsibilities to their best and use all available channels to disclose and establish their brand advantages. These challenges need urgent resolution.

In China, during the fight against the pandemic, SMEs used media channels to disclose and fulfill their internal corporate social responsibility (ICSR), earning employees’ praise and the recognition of consumers. Existing research on the impact of SMEs using we-media channels to disclose their ICSR on consumer behavior during epidemics is scarce. Previous research on the ICSR of enterprises is limited to employee satisfaction (Rosen and Levy 2013), employee civic behavior (Farooq et al. 2014), and other organizational internal behaviors. However, research on the spillover effects of external stakeholders is relatively lacking. Moreover, there are few studies on the externalization of ICSR. Based on the attribution theory, it is believed that performing external corporate social responsibility (ECSR) activities is altruistic while performing ICSR activities is self-interested (Jones et al. 2014). Therefore, only when a enterprise fulfills its ECSR and consumers have recognized the brand will the ICSR have a spillover effect (Glavas and Godwin 2013; Story and Neves 2015). In this study, we explain the mechanism of SMEs’ externalization of ICSR, under the impact of COVID-19. Additionally, with the maturity of social networking technology, SMEs can use low-cost and convenient we-media instead of traditional media to directly attract the public and interact with consumers. However, enterprises relied on annual reports and mainstream media to present their social responsibilities in the past. Most disclosures concerned enterprise economic responsibilities, environmental responsibilities, and public welfare donations (Katmon et al. 2019). There is little research on using we-media disclosures to fulfill employees’ ICSR.

This study focuses on the impact of COVID-19 on the increased use of we-media channels by SMEs. Additionally, we determine the impact of ICSR on consumer brand attitudes and the internal mechanism of SMEs. The internal mechanism between fulfilling ICSR and consumers’ external responses compensates for the separation of ICSR and external spillover effects, discussed in existing studies. Social responsibility has important theoretical value and practical implications for improving consumer brand attitudes.

## Literature review

### The impact of COVID-19 on SMEs

Existing research on the impact of catastrophic shocks such as epidemics on SMEs mainly includes the following. First, such shocks will cause certain material losses. Basker and Miranda (2014) use census data from the US and find that the survival conditions of enterprises are directly proportional to the material losses as a result of catastrophic shocks such as floods and earthquakes. Besides, the more serious the shock is, the higher the exit rate. Second, some catastrophes also affect the commercial property of SMEs, such as inventory and equipment. Further, SMEs’ order fulfillment and future sales are affected, an important reason for their bankruptcy or business recovery. Additionally, catastrophes such as epidemics impact the labor market and the upstream and downstream of the industrial chain. Therefore, the loss of employees and customers is an important factor affecting the recovery of enterprises (Corey and Deitch 2011). However, the impact of COVID-19 on SMEs has manifested in the uncertainty of the pandemic. The number of cases in developed economies in Europe and the US has been climbing; therefore, the global supply and industrial chains have been blocked. The uncertainty in SMEs’ operations has increased. Meanwhile, the decline in investors’ risk tolerance has led to financing difficulties for SMEs, increasing their liquidity and debt default risks, and intensifying the fluctuation in economic operation. The governments of various countries have strictly controlled the flow of people, inevitably leading to delays in labor supply and disruption of the original division of labor. The collapse of the supply system has broken the smooth transmission of the industrial supply chains, causing total supply and demand to regress. In summary, COVID-19 is special, and epidemic prevention measures such as lockdowm have effectively prevented and controlled its spread; however, the real economy has been temporarily suspended. SMEs are faced with operational difficulties and are concerned with surviving this crisis. Under the present epidemic situation, SMEs focus on saving themselves by attracting consumers and quickly converting their products into cash flow. Therefore, the process of SMEs fulfilling their CSR to gain consumer recognition and favor to in order to overcome this crisis is a challenge that all SMEs must tackle urgently.

### Internal and external CSR and disclosure channels

Bowen (1954) first define CSR as the pursuit of all activities consistent with social values; that is, out of sympathy for and empathy with society, entrepreneurs donate and help vulnerable groups. Mcelhaney (2009) believe that CSR is a spontaneous behavior of enterprises. These behaviors or activities are not limited by a enterprise’s interests or legal requirements; rather, they align with current social norms, values, and expectations. The existing research on CSR is based on large enterprises with rich resources (Block and Wagner 2014). However, SMEs with relatively scarce resources cannot apply the research conclusions of large enterprises. Mayson (2011) highlights that SMEs are not large enterprises; thus, the theory applied to large enterprises needs some modifications to meet the uniqueness, context, and logic of SMEs. When designing their CSR strategy, SMEs should focus on CSR activities that can enhance competitive advantage and promote enterprise growth (Wellalage et al. 2019). In both developed and developing countries, SMEs provide more than half of all job opportunities and make great contributions to the creation of national economic value.

There are different classifications of CSR. According to Jones et al. (2014), CSR can be divided into ICSR and ECSR. Turker (2009) highlights that ICSR is related to employees’ working environment, including physical and psychological protection, manifested in concerns about employee health and welfare, training and promotion, fair opportunities, and work–life balance. Regarding employee responsibility (ER), George and Bettenhausen (1990: pp. 698–709) indicate that “If managers want to work hard to cater to end customers, then they should first cater to internal customers (employees).” Hejjas et al. (2019) show that employees are an important object of enterprises’ social responsibility communication. ICSR is an important “internal marketing tool” for attracting and maintaining employees, affecting organizational fairness, trust, commitment, satisfaction, and employee civic behavior. ICSR activities generally affect only employee behavior. However, ECSR is related to local communities, business partners, suppliers, consumers, public institutions, nonprofit organizations, and the environment, including donations, voluntary activities, and environmental protection. Therefore, ECSR concerns consumer responsibility (CR) (Imran et al. 2016). Chen et al. (2008) find that ECSR is a marketing tool that attracts consumers and affects consumption. The impact on enterprises’ brand image, positive word-of-mouth (WOM) communication, purchase intentions, and customer loyalty by performing ECSR activities are recognized. ECSR affects only consumer behavior. ICSR and ECSR address different objects. Thus, they are bound to have different effects (Lee and Back 2008). Imran et al. (2016) further point out that the impacts of ICSR and ECSR involve two different paths. In summary, from the perspective of consumers, ECSR is “eternal focused,” and ICSR is “internal focused.” Previous research has discussed the impact of ECSR on external consumers, the impact of ICSR on internal employees, the impact of a small number of ECSR activities on internal employees, and the influence of segmentation on the relationship between ICSR and ECSR of an enterprise. Existing research is yet to uncover the specific transmission mechanism of the externalization of ICSR. Therefore, from the perspective of consumers, this study focuses on the mechanism of SMEs using media channels to fulfill and disclose their ICSR to strengthen their consumer brand attitudes.

CSR disclosure refers to the concept, strategy, and methods by which enterprises fulfill their CSR, the direct and indirect impacts, and the achievements and deficiencies caused by their business activities in the economic, environmental, and social fields. In other words, it involves the display and reporting of information (Stuart et al. 2020). The disclosure of CSR activities by large global enterprises, through CSR annual reports and mainstream media reports, has become a common practice (Muslu et al. 2019). The CSR report is an important nonfinancial information disclosure carrier and communication platform between the enterprise and stakeholders (Stuart et al. 2020). Voluntary CSR disclosures directly affect the public’s perception of enterprise image, thereby enhancing consumer brand attitudes (Chen et al. 2016). Enterprises that actively disclose their CSR generate a positive reputation, and when they are confronted by negative events, such CSR disclosures reduce negative reactions of investors (Christensen 2016). Since the twenty-first century, Internet technology has rapidly developed worldwide, accompanied by the advent of the era of we-media, which has subverted previous forms of media. We-media refers to ways in which members of the public can publish their facts and news through the Internet and other channels, such as Weibo, public accounts, and Twitter (Shayne and Chris 2003). Due to the inexpensive and convenient nature of we-media, any information may be widely spread on social networks, forming public opinion hot spots. The rise of we-media has gradually eroded traditional media. Due to the influence of public opinion, many events are first disclosed in we-media, followed by reports on traditional media, ultimately causing a widespread impact. Therefore, we-media constitutes an opportunity for SMEs. Due to their lack of funds and resources, SMEs can use inexpensive we-media to disclose their CSR behavior and interact with the public more effectively, instead of the previous intermediation process of traditional media.

### Consumer brand attitudes

Attitude is a combination of three parts: cognition (belief), emotion (feeling), and behavior (reaction tendency) (Yoon and Park 2012), including overall positive or negative evaluations of enterprises, brands, products, among others. Attitude can include likes or dislikes, passionate feelings, and behavioral tendencies (Kotler and Keller 2006). Brands help consumers differentiate the quality of products or services between enterprises, which can increase the value additions of goods, bring higher product profits and make products unique in the minds of customers (Park et al. 1986). Therefore, brand attitudes refer to consumers’ overall evaluation of a particular brand (Yoo and MacInnis 2005). When consumers make purchase decisions, they evaluate a brand and judge whether the attributes are positive or negative, reflecting the brand attitude (Jung and Seock 2016). However, the brand attitude itself is affected by factors including consumer-side factors and brand-side factors. Consumer-side factors include self-concept, consumer knowledge, and internal driving factors. For example, the entire process of the consumer self-concept life cycle affects brand attitudes (Gonzalez-Jimenez 2017). Consumers’ perceptions of brands lead to brand differentiation strategies that produce diametrically opposite results in different markets (Melania et al. 2018). The internal drivers of enterprise employees can affect their evaluation of the brand as well (King et al. 2017). Factors on the brand side include the brand name and the brand’s presentation platform. For example, different personal pronouns in the brand name (first vs. second vs. third person) will affect consumers’ decision-making preferences for branded products (Kachersky and Carnevale 2015). The presentation of brands on different social networking sites affects consumer attitudes (Mukherjee and Banerjee 2019). Kotler and Keller (2006) also mention that when consumers show a positive attitude toward a brand, the possibility of using the brand’s products increases; conversely, when consumers have a poor attitude toward a brand, the possibility of using the brand’s products decreases. When consumers make purchase decisions, they will maximize their perceived interest in a brand and use it as the basis for purchase based on their preferences (Chompunuch and Rian 2000). Wong and Merrilees (2008) define consumer brand attitude as a key factor in measuring whether a brand is successful in the market. Lee and Back (2008) divide consumer brand attitudes into three aspects: WOM, brand commitment, and brand image (general brand impressions). In summary, this study refers to the studies discussed and selects three aspects, i.e., new customers’ willingness to try new products, old customers’ repeated purchases, and positive WOM communication in considering consumer brand attitudes.

## Study 1

Previous studies on CSR have focused on first-hand questionnaire survey data; that is, researchers preset the influencing factors and then supplement the research with a questionnaire survey designed for data collection (Glavas and Godwin 2013). This method is affected by the social desirability bias of completing the questionnaire, impacting the objectivity of the research results. Thus, the current new trend of big data and web text content analysis is used to investigate consumer behavior. This method has the advantage of analysis based entirely on real and objective data. Therefore, we use content analysis to explore the impact of ICSR disclosure of SMEs on consumer brand attitudes.

### Data source

We select SMEs in the catering industry, fulfilling their ICSR as typical cases. Specifically, on February 8, 2020, a video was published by the restaurant chain Laoxiang Chicken through their Weibo. In less than 10 mins, 10 million people have seen the video. In the video, Shu Congxuan, chairman of Laoxiang Chicken’s board of directors, said, “Laoxiang Chicken is conservatively estimating a loss of at least 500 million yuan. A few days ago, I received this joint letter. It said that during the epidemic period, you employees would voluntarily give up your wages, and all of you signed and pressed your fingerprints on it. I think you are confused. Even if we have to sell our houses and cars, we should do everything possible to ensure that you have food and work!”

### Comments on the crawler analysis process

On February 28, 2020, we use a Python-based data crawler to capture the comments on the Weibo “Just now, the chairman of Laoxiang Chicken tore up the joint letter of employees,” originally published on February 8, 2020, on the official Laoxiang Chicken website, capturing 14,123 comments. The fuzzy classification method is used to verify the impact of SMEs’ ICSR on consumer brand attitudes.

First, we need to remove duplicates, filter out comments that are not meaningful, and filter out comments that are too short to be analyzed, such as “1” and “OK!” as well as comments that are not related to the research content, such as “fat and common.” Next, the comments are segmented, and the nouns, verbs, adjectives, and adverbs in each phrase are segmented. However, the phrases without verb-object relationships are not segmented. We use the ICTCLAS word segmentation tool of the Chinese Academy of Sciences for large-scale word segmentation and long, complex sentences. Then, by compiling a marketing dictionary for the comments (see Table 1 for the specific contents of the marketing dictionary), the reliability of the coding results is determined by the interrater reliability of three independent coders. Inconsistencies are discussed by the entire research team and resolved through consultation with senior marketing professors.

**Table 1:** Specific marketing dictionary content

Marketing dictionary dimension	Words corresponding to the dimensions of the marketing dictionary
Early adopters	I really want to try it, I want to try it, I have to eat it once, when to come to xx to open a store, does xx have a Laoxiang Chicken, hurry to settle in, and if you encounter it, you must buy it...
Willingness to repurchase	I want to eat the name of xxx dishes, I have wanted to eat them for a long time, I went to eat immediately after the outbreak, eat a few times, take out daily, and later eat chickens to eat Laoxiang Chickens, xxx dishes like delicious …
Positive WOM	Touched, awesome, pink, good, good business, crazy likes to forward, tell people around, great, choose Laoxiang Chicken when you order takeout …
Entrepreneurship	Entrepreneurship, humor, down-to-earth, capable, lovely chairman, especially handsome …
Job search intentions	Envious of the employees, are you still short of staff, are there school recruits, the benefits are really good, the salary is good, the employees are happy …

Finally, we enter the nouns and noun phrases into the evaluation system, use recursive methods to measure the weights of each level, and obtain the score level of each feature level. The number of occurrences and weights are used to calculate the score level of the first-level dimension. The total number of occurrences is then used to calculate the first-level construct from the relevant information of the second-level construct to obtain the overall evaluation result.

### Weight calculation model

The fuzzy analytic hierarchy process effectively addresses the inaccurate index values and transforms qualitative evaluations into quantitative evaluations. The calculation model of the average score of a single feature value is as follows: The score of all users (*n* users) with score feature *i* is *x*_*i*_, *x*_*i*2_ … *x*_*in*_. Then, the average score *X*_*i*_ of feature *i* is as follows:

1 $$ \overline{X_i}=\frac{\sum \limits_{j=1}^n{X}_{ij}}{n}. $$

The average score of a single second-level construct is calculated as follows: Suppose all features (*m* features) under a certain second-level construct *k* are *x*_*k1*_, *x*_*k*2_… *x*_*km*_, and the number of times the *m* features are mentioned is *p*_*k1*_, *p*_*k*2_… *p*_*km*_, respectively. Then, the average score of the second-level construct *k*, $\overline{Y_k}$ is as follows:

2 $$ \overline{Y_k}=\frac{p_{k1}\kern0.28em {x}_{k1}+{p}_{k2}\kern0.28em {x}_{k2}+\cdots +{p}_{km}{x}_{km}}{p_{k1}+{p}_{k2}+\cdots +{p}_{km}}=\frac{\sum \limits_{j=1}^m{p}_{kj}{x}_{kj}}{\sum \limits_{i=1}^m{p}_{ki}}. $$

The average score of a single first-level construct is calculated as follows: Suppose all features (*g* second-level constructs) under a first-level construct *r* are *y*_*r1*_, *y*_*r2*_ …*y*_*rm*_ and the number of times the *m* features are mentioned is *q*_*r1*_, *q*_*r2*_…*q*_*rm*_, respectively. Then, the average score of first-level construct *r*, $\overline{z_r}$ is as follows:

3 $$ \overline{Z_r}=\frac{q_{r1}\kern0.28em {y}_{r1}+{q}_{r2}\kern0.28em {y}_{r2}+\cdots +{q}_{rm}{y}_{rm}}{q_{r1}+{q}_{r2}+\cdots +{q}_{rm}}=\frac{\sum \limits_{j=1}^m{q}_{rj}{y}_{rj}}{\sum \limits_{i=1}^m{q}_{ri}}. $$

In this study, the application steps of fuzzy hierarchical evaluation are as follows: First, we determine the evaluation item set *F = (f*_*1*_*, f*_*2*_*, f*_*3*_*, f*_*4*_*, … f*_*n*_*),* from which we construct *F = (brand attitude, other behavior)*, *f*_*1*_ *= (a*_*1*_*, a*_*2*_*, a*_*3*_*, a*_*4*_ *… a*_*n*_*)*, *and f*_*1 External consumption behavior*_ *= (willingness to try, willingness to repurchase, positive WOM).* The product characteristic index is *f*_*1*_ *= (a*_*1*_*, a*_*2*_*, a*_*3*_*, a*_*4,*_ *… a*_*n*_*)* and *f*_*1 Internal organizational behavior*_ *= (entrepreneur image, employee benefits).* Then, the captured comments are processed through word segmentation to obtain nouns or motionless relation phrases. The similarity matching principle is then used to attribute the corpus to the characteristic word class. The related literature’s interpretation of each first-level dimension summarizes the feature words under the first-level dimension to construct the feature word lexicon using the marketing dictionary. Similarly, the score scale of each item is determined, and the score is refined, E = (I like it very much, I like it, Generally I do not like it, I do not like it very much), where 5 points means they like it very much and 1 point means they do not like it very much. The degree of adjectives can be specifically divided into the following categories: 5 = (very good, very happy, too satisfied, super-powerful …), 4 = (good, liked, satisfied …), 3 = (nice, OK …), 2 = (decent, OK, very fair …), 1 = (not good, really bad, terrible …). Finally, after counting each feature word’s frequency of occurrence in the corpus, based on the weight calculation model, the corresponding weight and score for each corpus are calculated.

### Data analysis

**Fig. 1:**
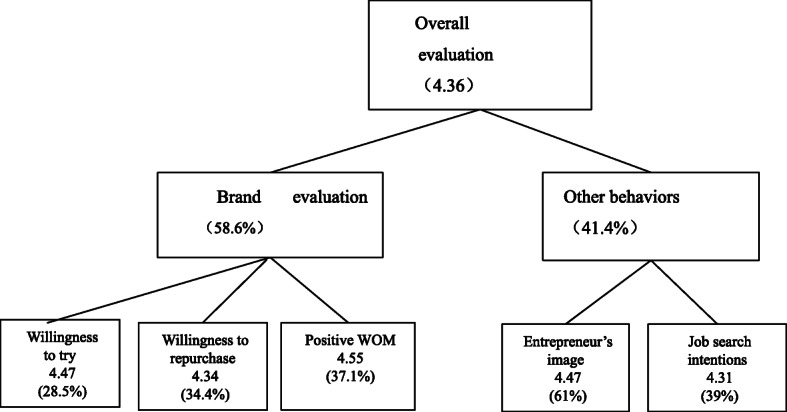
Event evaluation results

After calculating the frequency of 14,123 comments and obtaining the frequency of each feature word in the corpus, based on the weight calculation model, the corresponding weight and score of each corpus are calculated, as shown in Fig. 1 below.

The analysis results in Fig. 1 show that the final comprehensive score of the event is 4.36. The consumer comments mostly involve consumer brand attitudes (58.6%), while there are relatively few comments on other behaviors (41.4%). In terms of brand attitudes, consumers show a high positive reputation (4.55), while for other behaviors, consumers show a high rating for the entrepreneur’s image (4.47).

Table 2 shows further analysis of the weights of the 14,123 reviews. Considering consumer attitudes, the sum of consumer brand attitudes (160.5%) is significantly higher than that of other behaviors (63.1%). Once the enterprise has fulfilled its ICSR, consumers show more positive attitudes toward enterprise brand recognition. Further analysis of consumer attitudes reveals that consumer brand attitudes are more likely to lead to repurchases.

**Table 2:** Proportion of consumer attitudes

Characteristic	Consumer brand attitude			Other behaviors	
Index	Early adopters	Will to repurchase	Positive WOM	Entrepreneurship	Employee benefits
Mentions	4857	6977	5125	5522	1025
Mentioned	45.3%	63.5%	51.7%	55.6%	7.5%

This study uses data crawler technology and a fuzzy comprehensive evaluation method to extract the evaluation information in consumer-generated content by semantic analysis and similarity matching of text comment data. The results show that SMEs fulfill their ICSR by using Weibo media disclosures during the epidemic, which produces positive evaluations and consumer brand attitudes (willingness to taste fresh food, repurchase intentions, and positive WOM). In this era of social networks, information spreads rapidly on the Internet, and consumers pay increasing attention to information about the activities of SMEs (Berger and Kanetkar 1995). In the case of the same products, for SMEs that actively fulfill their ICSR, consumer brand attitudes are improved.

Previous studies have found that the relationship between ECSR and ICSR is a moderating factor in spreading internal employees’ social responsibility to the outside, making employees responsible for obtaining different brand attitudes (Yoon et al. 2006). When the level of ECSR is high, the performance of both ICSR and ECSR indicates that enterprises not only protect the interests of “self-related” employees but also protect the interests of “other-related” consumers. Consumers believe that enterprises undertake full responsibility for all stakeholders consistently (Kelley 1971). Therefore, consumers attribute high ER to altruism, which is beneficial. When ECSR is low, there is a gap between the performance of ICSR and ECSR, the high level of ER shows uniqueness, and consumers attribute the behavior of the enterprise to self-interest. Kelley (1971) believes that enterprises are purely self-interested and ignore other responsibilities, thus weakening the positive impact of ER on brand attitude and presenting negative evaluations even with high ER performance. However, based on the findings of this study, SMEs fulfill only ICSR, which is recognized by the majority of consumers; that is, the process of the externalization of ICSR is realized, contradicting previous research conclusions. The primary reason for these contradictions is that in previous studies, it is only under the premise of fulfilling ECSR will the enterprise perform ICSR, and consumers will attribute the behavior of the enterprise to altruism. The root cause is that the enterprise’s ECSR involves a sacrifice of its interests to repay society. Consumers feel that the enterprise’s self-sacrifice will produce profits. During the epidemic, SMEs face great pressure to survive and are unable to fulfill ECSR. However, enterprises are willing to sacrifice their interests to protect the interests of employees even in the absence of profits. Therefore, consumers perceive the self-sacrifice of enterprises, leading to an altruistic attribution, improving consumers’ brand attitude.

Study 1 has the following limitations. (1) It verifies the main effect of SMEs fulfilling CSR to enhance consumer brand attitudes under the impact of the epidemic and does not explore or verify the intermediary mechanism of consumer perceptions of enterprise self-sacrifice. (2) In Study 1, we select the Weibo of Chinese SMEs as an example, and it is difficult to exclude the influence of consumer stereotypes on brand attitudes. It is also difficult to completely clarify the ECSR previously performed by Laoxiang Chicken enterprise and the spillover effect of the enterprise’s ICSR on improving consumer brand attitudes in events like the general manager of the enterprise tearing up employees’ joint letter. (3) Study 1 does not explore the boundary conditions that exist in the process of externalizing ICSR of SMEs under the impact of the epidemic. To compensate for the above limitations, we further explore this issue in Study 2.

## Study 2

The purpose of Study 2 is to effectively eliminate the interference of consumers in the inherent image of enterprises, the perception of enterprise scale, and the ECSR. By fabricating material used by SMEs on Weibo to disclose their ICSR under the impact of the epidemic, a structural equation model is constructed using the questionnaire survey method. This method is advantageous as it can effectively control the experimental process and factors, clarify the causal relationship between core variables, verify the impact of SMEs’ ICSR on consumer brand attitudes during the epidemic, verify the mediating role of perceived enterprise self-sacrifice between enterprises’ ICSR and consumer brand attitudes, and address the moderating effect of the perceived degree of enterprise losses.

### Hypothses and model

#### Self-sacrifice theory

Self-sacrifice theory refers to individuals’ willing to bear losses to adhere to their beliefs and values ​(Yorges et al. 1999). Existing research on sacrifice mostly focuses on leaders’ sacrificial behavior (Iacobucci 2017). To achieve its goals and mission, organizations are not afraid of personal interest losses and actively contribute to the interests of the organization. Employees’ perception of leaders’ sacrifice is related to their evaluation of leaders (de Cremer and van Knippenberg 2004). De Cremer and van Knippenberg (2004) explore the relationship between a leader’s sacrifice and charisma by manipulating context questionnaires. The results show that a leader’s sacrifice is positively correlated with their perceived charm. Arnold and Loughlin (2010) find that leaders’ self-sacrifice is positively correlated with employees’ evaluation of leadership effectiveness. The higher degree of leaders’self-sacrifice rated by employees, the higher their leadership effectiveness. De Cremer and van Knippenberg (2004) target 118 enterprise management departments. In-service students measure the degree of sacrifice of existing work leaders and their work efficiency. The study find that employees assess the degree of self-sacrifice and effectiveness, which shows a significant positive correlation. The higher the employee’s self-sacrifice, the higher the employee’s perceived self-efficacy. Therefore, self-sacrificing leadership can promote civic organizational behavior, interpersonal help, cooperation, reciprocal behavior, and work participation (Cleo 2016). However, there is still little research on the relationship between consumer perceptions of enterprise self-sacrifice and consumer behavior. Therefore, this study uses self-sacrifice theory to explain enterprise performance under COVID-19 and the internal mechanism of the impact of ICSR on consumer brand attitudes. Consumer self-sacrifice is defined as the perception that a enterprise is willing to bear losses to protect the interests of employees and consumers.

#### The impact of ICSR on consumers’ perceived enterprise self-sacrifice

Studies such as Turker (2009) have found that enterprises fulfilling ICSR in treating employees significantly impact consumers’ purchase intentions. Studies such as Smidts et al. (2000) find that with the increase in news channels, enterprises’ performance concerning ER can be spread via online media, forums, TV, among others, and be known by consumers, affecting brand attitudes. When enterprises care for their employees, consumers also trust them and are assured of product quality. Consumers tend to trust and buy products by those enterprises that actively take on CSR (Jin and Lee 2019; Thomas et al. 2016). Therefore, we believe that SMEs affected by COVID-19 have taken the initiative to bear ICSR despite huge losses. Consumers believe that enterprises have suffered great economic losses. However, under the impact of the epidemic, although some enterprises did not implement layoffs, it is still necessary to protect employees’ salaries. Enterprises are willing to care for and pay attention to the interests and needs of organizational members. Additionally, they are willing to maintain the well-being of organizational members and sacrifice the enterprise’s interests (Ruggieri and Abbate 2013). Therefore, consumers believe that the enterprise is ethical; thus, willing to spread the WOM of the brand, and new customers will be willing to try and buy products. Old customers will increase customer loyalty and be willing to buy the brand’s products repeatedly. Accordingly, the following hypothesis is proposed.

- *Hypothsis 1: Under the impact of COVID-19, SMEs’ fulfillment of ICSR positively affects consumers’ perceived enterprise self-sacrifice.*

#### The impact of consumers’ perceived enterprise self-sacrifice on consumer brand attitudes

Under the impact of the epidemic, SMEs have suffered a huge blow; however, they insist on fulfilling their ICSR to protect employees’ basic living. Consumers perceive that enterprises have made self-sacrifice and show a high degree of commitment to their employees; the spirit of responsibility results in a spillover effect. It makes consumers think that the enterprise’s brand is more trustworthy and produces purchase intentions and positive WOM communication. According to social learning theory, consumers perceive the enterprise’s spirit of self-sacrifice, demonstrating that the enterprise meets a high ethical and moral standard (de Cremer and van Knippenberg 2004). This prompts consumers to regard the enterprise as a role model worth emulating, resulting in an emotional attachment to the role model enterprise (Mulder and Nelissen 2010) and preferences for the enterprise. Accordingly, Hypothesis 2 is proposed.

- *Hypothesis 2: Consumers’ perceived enterprise self-sacrifice positively affects consumer brand attitudes.*

#### The impact of consumers’ perceived degree of enterprise losses

**Fig. 2:**
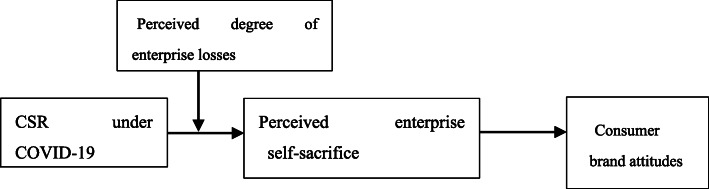
Conceptual model

Story and Neves (2015) find that after enterprises fulfill their CSR, consumers judge whether they are self-serving or altruistic. Consumers develop positive brand attitudes toward CSR behaviors motivated by altruism and perceive high product quality. In contrast, they develop negative brand attitudes toward self-motivated CSR behavior and perceive low product quality. Compared with ECSR (such as donations and public welfare), ICSR is usually attributed by consumers to self-interest (Carroll and Shabana 2010). Glavas and Godwin (2013) believe that only when a enterprise considers both ICSR and ECSR, will consumers develop a sense of identification with the enterprise, thereby enhancing the willingness to buy their products. However, for enterprises only implementing ICSR, consumers reduce their willingness to buy brands due to the self-interest of the enterprises. In summary, we believe that in different industries, SMEs have suffered different losses as a result of COVID-19. When consumers perceive that under severe losses due to the epidemic, the enterprise itself is in jeopardy and would rather sacrifice itself and perform ICSR to protect employee welfare, they make an external altruistic attribution. Consumers believe that the enterprise has noble ethics, thereby enhancing consumer brand attitudes. Due to their limited resources, the survival of SMEs during the epidemic is worrying, but so is the survival of the employees, which demonstrate the noble ethics and sense of responsibility of the enterprise. However, when consumers feel that the losses suffered by the enterprise under COVID-19 are not serious and that the enterprise fulfills only its ICSR and not the corresponding ECSR, consumers will make an internal self-interest attribution and believe that the enterprise may even be suspicions of “making a show,” thus reducing consumer brand attitudes. Accordingly, Hypotheses 3 and 4 are proposed, and the overall model is shown in Fig. 2.

- *Hypothsis 3: Regarding the perceived degree of enterprise losses under COVID-19, SMEs’ fulfillment of ICSR will have a positive moderating effect on the impact of the perceived enterprise self-sacrifice.*
- *Hypothsis 4: The perceived degree of enterprise losses will strengthen the perceived enterprise self-sacrifice under COVID-19.*

### Research design

#### Sample and data collection

In this study, we use the virtual situation survey method followed by the situation questionnaire to let the subjects read the survey scenario and then fill out the questionnaire. The survey target is the consumer group. The participants need to read the following information in the situation manipulation part: The enterprise’s Weibo and public account platforms issued announcements. Affected by COVID-19, the enterprise conservatively estimated losses of at least 30 million. However, the enterprise promised not to lay off employees during the epidemic, and the wages of employees in self-isolation would be paid, even if the enterprise had to sell its houses or cars. Every effort would be made to ensure that employees had food and work. Following relief from the epidemic, the enterprise must resume business on the premise of ensuring employees’ safety. After reading the survey scenario materials, the subjects continue to fill out the questionnaire. The WeChat group collect questionnaires by snowballing. A total of 975 questionnaires are recovered. The information of the test samples is shown in Table 3. We eliminate incomplete and invalid questionnaires and obtain 946 valid responses. The effective response rate is 97.03%. We investigate whether there is a reaction bias in the sample and perform an independent-sample *t*-test on the sample’s descriptive statistics. The results show *t* > 0.05, indicating that the covariates (gender, outbreak area, education, income, age and type of work) are not significantly different, meaning that the sample questionnaire has no response bias.

**Table 3:** Descriptive statistics of the research samples

Sample characteristics	Classification criteria	Sample		Sample characteristics	Classification criteria	Sample	
		quantity	Percentage			quantity	percentage
Gender	Male	514	54.3	Income	1–3000 yuan	155	16.4
	Female	432	45.7		3001–6000 yuan	354	37.4
Outbreak area	Areas with more than 20,000 diagnoses	455	48.1		6001–9000 yuan	220	23.3
	Areas with between 10,001 and 20,000 diagnoses	285	30.1		More than 9000 yuan	217	22.9
	Areas with 5000 to 10,000 diagnoses	174	18.4	Type of work	Student	298	31.5
	Areas with less than 5000 diagnoses	32	3.4		Teacher	136	14.4
Education	High school	355	37.5		Government civil servant	187	19.8
	Undergraduate	334	35.3		Enterprise or institutional worker	191	20.1
	Master’s degree	174	18.4		Freelancer	134	14.2
	PhD	83	8.8				

Given the context of this study, we use a more mature scale. Additionally, there are discussions among scholars to make appropriate amendments to the scale. The research scale used by Glavas and Godwin (2013) is adopted to measure whether the enterprise performs ICSR during the epidemic. To measure perceived enterprise self-sacrifice, we adopt the scale used by de Cremer and van Knippenberg (2004). To measure, we adopted the scale presented in (de Cremer and van Knippenberg 2004). We apply Lynn (1982) scale to the research question of this paper. The scale is translated, and the items are appropriately added and changed. The specific measurement items are shown in Table 4. The five variables discussed are measured by a 7-point Likert scale, where 1 means “completely disagree,” and 7 means “completely agree.” In this study, we select gender, outbreak area, education, income, age and type of work as covariates to avoid the influence of other control variables; the gender and type of work variables are treated as a dummy, and the other variables are treated as continuous variables.

**Table 4:** Variable measurement item list

Measuring variable	Measurement item	References	Factor loading	CR	Scale reliability
Under the impact of the epidemic, the enterprise fulfills its ICSR	The enterprise values the safe working environment of its employees	Glavas and Godwin (2013)	0.854	0.922	Cronbach’s *α* = 0.924
	The enterprise provides employees with job opportunities requiring valuable skills and talent		0.796		
	The enterprise protects the rights and interests of employees		0.921		
	The enterprise gives employees a sense of security		0.884		
Perceived enterprise self-sacrifice	The enterprise sacrifices its interests for the benefit of employees	de Cremer and van Knippenberg (2004)	0.751	0.835	Cronbach’s *α* = 0.884
	To protect employees, the enterprise has taken a risk		0.866		
	The enterprise helps employees solve difficulties, even at the expense of its interests		0.758		
Perceived degree of enterprise losses	The enterprise is affected by the epidemic and suffers a great loss in performance	Lynn (1982)	0.841	0.896	Cronbach’s *α* = 0.933
	The enterprise is affected by the epidemic, and its survivability is greatly challenged		0.853		
	The enterprise is affected by the epidemic, and it will be very difficult to resume production		0.889		
Consumer brand attitudes	I am willing to pay a higher price to buy products (services) of the enterprise’s brand	de Cremer and van Knippenberg (2004)	0.798	0.943	Cronbach’s *α* = 0.917
	If I have not been in contact with the enterprise’s brand before, I’m now willing to try to buy the enterprise’s branded products (services)		0.924		
	I am willing to continue buying products (services) of the enterprise’s brand in the future		0.933		
	I am willing to recommend the enterprise’s branded products (services) to others		0.927		

#### Reliability and validity analysis

First, we confirm the reliability and validity of the questionnaire, followed by the hypothesis test. According to the values in Table 4, the factor loading of all items in this study are above 0.7, indicating that they will have a significant impact on potential variables. The composite reliability (CR) of the variables is also higher than 0.7; thus, each construct index has a high degree of internal consistency. Additionally, according to the correlation coefficient table (see Table 5), the average variance extracted (AVE) values of each construct for measuring the discriminant validity are all greater than 0.5. Furthermore, the square root of each potential variable’s AVE is greater than the correlation coefficient of other potential variables, and the results show that the research model has good discriminant validity.

**Table 5:** Variable correlation coefficient table

Variable	Mean	Standard deviation	1	2	3	4	5	6	7	8	9	10
1. ICSR	5.41	0.55	(0.75)									
2. Perceived enterprise self*-*sacrifice	4.69	0.97	0.38**	(0.63)								
3. Perceived degree of enterprise losses	5.15	0.57	*−*0.19	0.64**	(0.74)							
4. Consumer brand attitudes	5.36	0.60	0.26**	0.17	0.34**	(0.81)						
5. Gender	1.56^a^	0.50	*−*0.17	*−*0.10	*−*0.14	*−*0.20	–					
6. Age	35.01	100.69	*−*0.15	*−*0.08	*−*0.12	*−* 0.22	*−*0.06	–				
7. Education	1.37^b^	0.46	0.09	0.15	0.20	0.06	0.18	0.27**	–			
8. Income	1.41^c^	0.51	0.04	0.13	0.21	0.07	0.18	0.22**	0.09	–		
9. Outbreak area	2.04^d^	0.88	0.17	0.11	0.26**	0.07	0.12	0.07	0.09	0.15	–	
10. Type of work	0.52^e^	0.46	*−*0.19	*−*0.12	*−*0.15	*−*0.24*	*−*0.06	*−*0.18	*−*0.11	*−*0.16	*−*0.20	–

*Notes. n* = 946; AVE values are in parenthesis. ^a^ 1 = male, 2 = female; ^b^ 0 = high school, 1 = undergraduate, 2 = master’s degree, 3 = PhD; ^c^ 0 = 1–3000 yuan, 1 = 3001–6000 yuan, 2 = 6001–9000 yuan, 3 = more than 9000 yuan; ^d^ 0 = area with less than 5000 diagnoses, 1 = area with 5000 to 10,000 diagnoses, 2 = area with 10,001 to 20,000 diagnoses, 3 = area with more than 20,000 diagnoses; ^e^ 0 = student, 1 = teacher, 2 = government civil servant, 3 = enterprise or institutional worker, 4 = freelancer

*Notes.* * *p* < 0.05; ** *p* < 0.01; *** *p* < 0.001, two-tailed test

#### Common method bias test

In this study, the questionnaire survey method is mainly used. The same subject provides multiple variable data. As there may be an effect of common method bias, we use Harman’s single factor test. Whether the enterprise fulfills its ICSR, perceived enterprise self-sacrifice, perceived degree of enterprise losses, and consumer brand attitudes are used to construct a single-factor structural equation model and to test the fit. The fit of the single-factor model is not ideal: *χ*^2^/*df* = 354.01, RMSEA = 0.09, SRMR = 0.06, CFI = 0.92, IFI = 0.533; to a certain extent, the common method bias in the measurements of this study is not serious.

#### Test results

We use AMOS 19.0 to construct a structural equation model to test the hypotheses: *χ*^2^/*df* = 2.38, GFI = 0.950, NFI = 0.938, TLI = 0.945, CFI = 0.921, and RMSEA = 0.070. These indicators test the fit of the model. The sample fit indicators are as follows: *χ*^2^/*df* between 1 *and* 3; the GFI, NFI, TLI, and CFI values are greater than 0.85; and the RMSEA less than 0.08, indicating that the research model has a good fit and can be used for further hypothesis testing. According to the test results of the standardized path coefficients in the model, the enterprise’s performance of ICSR under the impact of the epidemic is associated with the perceived enterprise self-sacrifice. The self-sacrifice has a positively significant impact (*β* = 0.71, *p* < 0.01). The enterprise’s ICSR under the influence of the epidemic positively impacts consumer brand attitudes (*β* = 0.48, *p* < 0.01). Therefore, perceived enterprise self-sacrifice is associated with consumer brand attitudes. The positive effect of attitudes is significant (*β* = 0.51, *p* < 0.01). Additionally, to further verify the stability of the moderating role of perceived enterprise self-sacrifice, we refer to Hayes (2013) and use Model 4 in PROCESS for verification. The results show that perceived enterprise self-sacrifice has a significant moderating role with moderating effect value of 0.198, in the enterprise’s ICSR and consumer brand attitudes during the epidemic (LLCI = 0.137, ULCI = 0.291, excluding 0). Therefore, Hypothese 1 and 2 are supported.

Further, we test the moderating effect of the perceived degree of enterprise losses. The results show that the interaction terms of the enterprise’s ICSR and the perceived degree of enterprise losses under the impact of the epidemic have a significant positive affect on perceived enterprise self-sacrifice (*β* = 0.10, *p* < 0.05). As shown in Table 6, Hypothsis 3 holds.

**Table 6:** The results of the hierarchical regression analysis of the moderating effect of the perceived degree of enterprise losses

Variable	Perceived enterprise self-sacrifice			
	Model 1	Model 2	Model 3	Model 4
Constant	4.76** (0.35)	4.62** (0.33)	4.61** (0.33)	4.63** (0.32)
Gender	*−*0.52 (0.43)	*−*0.13 (0.41)	*−*0.22 (0.42)	*−*0.21 (0.42)
Age	*−*0.24 (0.46)	*−*0.07 (0.40)	*−*0.04 (0.43)	*−*0.05 (0.42)
Education	0.09 (0.45)	0.21 (0.42)	0.24 (0.42)	0.18 (0.42)
Income	0.11 (0.44)	0.32 (0.41)	0.27 (0.41)	0.29 (0.41)
Outbreak area	0.19 (0.43)	0.05 (0.43)	0.03 (0.40)	0.04 (0.39)
Type of work	*−*0.26 (0.45)	*−*0.37 (0.48)	*−*0.48 (0.49)	*−*0.39 (0.48)
ICSR (*x*_1_)		0.72 (0.19)**	0.60 (0.24)**	0.71 (0.20)**
Perceived degree of enterprise losses (*x*_2_)			0.17 (0.22)**	
*x*_1_ × *x*_2_				0.10 (0.04) *
*R*^2^	0.06	0.19*	0.20*	0.44*
△*R*^2^		0.13**	0.13**	0.10*
△ *f*		12.52**	6.45**	3.37*

*Notes.* * *p* < 0.05; ** *p* < 0.01; *** *p* < 0.001, two-tailed test

**Fig. 3:**
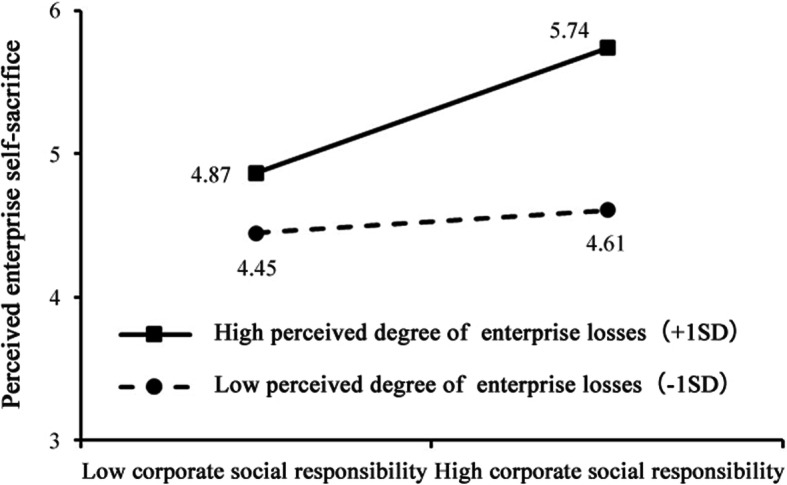
Diagram of the moderating effect of perceived degree of enterprise losses

We further analyze the moderating effect of perceived degree of enterprise losses and add a standard deviation to the mean of the variables to divide the sample into a enterprises groups with perceived high degree of loss and perceived low degree of loss. The study finds (as shown in Fig. 3) that the impact of a high degree of perceived enterprise losses on perceived enterprise self-sacrifice is significantly higher than the impact of a low degree of perceived enterprise losses, once again verifying Hypothesis 3.

We strengthen the moderated mediation effect of perceived enterprise self-sacrifice between the enterprise’s ICSR and consumer brand attitudes under the impact of the epidemic. The bootstrapping method is proposed, according to Edwards and Lambert (2007).

Based on Table 7, under the impact of the epidemic, when the level of perceived enterprise losses is low, the ICSR of enterprises has no significant effect on perceived enterprise self-sacrifice (*r* = 0.17, *p* > 0.05); when the level of perceived enterprise losses is high, the fulfillment of ICSR by enterprises has a significant positive effect on perceived enterprise self-sacrifice (*r* = 0.67, *p* < 0.01); the difference between the two is significant (Δ*r* = 0.50, *p* < 0.05). At the same time, in a enterprise with a low degree of perceived losses, the indirect impact of the enterprise’s ICSR and perceived degree of losses on consumer brand attitudes (through perceived enterprise self-sacrifice) under the impact of the epidemic is not significant (*r* = 0.12, ns), while in the case of an enterprise with a high degree of perceived losses, there is a significant positive effect (*r* = 0.95, *p* < 0.01), and the difference between the two is also significant (Δ*r* = 0.83, *p* < 0.01). Therefore, Hypothsis 4 is verified.

**Table 7:** Moderated mediation effect test

Moderator	Under the impact of the epidemic, the enterprise fulfills its ICSR (*X*) → perceived enterprise self-sacrifice (*M*) → consumer brand attitudes (*Y*)				
	Stage effect				
	The first stage *P*_*MX*_	The second stage *P*_*YM*_	Direct effect *P*_*YX*_	Indirect effect *P*_*YM*_ *P*_*MX*_	Total effect *P*_*YX*_ + *P*_*YM*_ *P*_*MX*_
High perceived losses	0.67**	1.38**	0.57	0.95**	1.52***
Low perceived losses	0.17	0.63	0.87	0.12	0.99
Difference	0.50*	0.75	*−*0.30	0.83**	0.53*
95% confidence interval	[0.05, 0.90]	[*−*0.30, 1.92]	[*−*1.90, 1.57]	[0.14, 1.75]	[*−*0.20, 1.25]

*Notes. P*_*MX*_ represents the independent variable → the influence coefficient of the intermediary variable, *P*_*YM*_ represents the intermediary quantity → the influence coefficient of the dependent variable, *P*_*YX*_ represents the independent variable → the direct effect of the dependent variable, *P*_*YM*_ *P*_*MX *_ represents the independent variable → indirect effect of the dependent variable, *P*_*YX*_ + *P*_*YM*_ *P*_*MX*_ represents the independent variable → the total effect of the dependent variable

* *p* < 0.05, ** *p* < 0.01, *** *p* < 0.001

Previous studies indicate that only when enterprises consider both ICSR and ECSR can consumers make external altruistic attributions to the performance of ICSR, thus enhancing brand purchase intentions (Glavas and Godwin 2013). We find that under COVID-19 epidemic, SMEs have improved their brand attitudes by using Weibo media to fulfill and disclose their ICSR activities. This is because consumers perceive the self-sacrifice of enterprises and believe that enterprises have noble morality and face great losses under the influence of the epidemic. Therefore, consumers are influenced by the spillover effects and are willing to buy the products (services) of these enterprises. This shows that COVID-19 epidemic falls within the performance of enterprises. The spillover effects also play a catalytic role in the process of CSR externalization, enriching and complementing research on the CSR externalization of SMEs from the perspective of consumers. Besides, this study verifies that when consumers perceive that the losses of enterprises performing ICSR under the influence of the epidemic are small, they attribute the ICSR fulfillment to the enterprise’s internal self-interest. Protecting employees’ interests is not a performance of enterprise self-sacrifice but the responsibility of enterprises to start work; thus, consumer brand attitudes will not be improved. When enterprises’ losses are small, and consumers do not perceive the self-sacrifice of enterprises, they will attribute the behavior of enterprises to their internal responsibility. This conclusion is consistent with previous research (Glavas and Godwin 2013). However, only when consumers perceive that enterprises fulfilling their ICSR have suffered will they make external altruistic attributions. Due to the limited resources of SMEs, they are unable to fulfill their ECSR. However, enterprises can sacrifice their interests to protect the survival of employees in the short term, sufficient to prove the sense of responsibility of enterprises. Therefore, consumers recognize these enterprises and are willing to help them resolve their difficulties by purchasing their products (services), further proving the catalytic role of COVID-19 epidemic.

## Research conclusion and discussion

### Theoretical contributions

This study makes a positive theoretical contribution to the relevant literature on CSR under COVID-19 epidemic.

First, although some studies have explored the impact of catastrophic (earthquakes, flash floods, among others) shocks on the operation of SMEs (Corey and Deitch 2011), COVID-19 has unique characteristics, primarily due to its long duration and large coverage area. Although epidemic prevention measures such as lockdown have been effective in preventing its spread, they have also caused SMEs to suspend their operations to a certain extent. SMEs have faced operational difficulties in the past, but now, they face a crisis for survival. Thus, it is difficult to apply the conclusions of previous epidemic research to COVID-19 situation. Besides, previous studies, from the perspective of SMEs fulfilling their ICSR, that explore ways to change the situation of SMEs under an epidemic are relatively scarce. Therefore, in the context of COVID-19, from the perspective of SMEs fulfilling their ICSR, this study explores the impact of the ICSR disclosure of SMEs on consumer brand attitudes, which enriches the research results on enterprise crisis management.

Second, this study proposes and demonstrates the mediating role of perceived enterprise self-sacrifice. On the one hand, previous research on self-sacrifice has focused on the impact of leaders’ self-sacrifice on organizational behavior (de Cremer and van Knippenberg 2004; Jin and Lee 2019; Mulder and Nelissen 2010; Thomas et al. 2016) and the impact of self-sacrifice on partner relationships in the field of social psychology (Chantal and Mark 2003). Regarding the impact of attitudes, this study has enriched the research results of the self-sacrifice theory. On the other hand, it has broadened the theoretical research perspective on the post-disaster psychological variables of social responsibility in the industry. In previous studies on CSR, scholars have already discussed the psychological consequences of perceived social responsibility (Farooq et al. 2014), enterprise identity (Story and Neves 2015), among others. However, the ECSR of SMEs is mostly examined under major public events. The psychological mechanism of change has not attracted the attention of scholars. This study is based on the theory of self-sacrifice. Further, it verifies that under COVID-19, SMEs can actively fulfill their ICSR. Consumers perceive enterprise self-sacrifice and believe that enterprises have a noble morality and sense of responsibility, resulting in spillover effects that enhance consumer brand attitudes. This finding promotes the psychological consequences of SMEs’ fulfillment of ICSR. It is also a theoretical extension and innovation based on self-sacrifice literature.

Third, the moderating role of consumers’ perceived degree of enterprise losses in SMEs’ fulfillment of ICSR under COVID-19 is systematically discussed. The degree of effort required to improve the situation has a very close relationship with an enterprise’s motivation to fulfill its ICSR. However, the theoretical discussion of previous research on this research structure is still limited. We find that perceived degree of enterprise losses plays a positive role in moderating the impact of SMEs’ ICSR on consumer brand attitudes under COVID-19. Only when consumers believe that enterprise losses are large will external altruism be attributed to the enterprise, thereby enhancing brand attitudes.

### Practical contributions

Brands with a high reputation (including consumer brand attitudes) and scaling the commanding height of enterprise market competition are the focus. Therefore, the practical contributions of this study mainly concern enterprises at the top level.

First, fulfilling ICSR is a rational choice for SMEs to find a new way out of difficulties. In the Internet era, the dispersal of information has changed, and the boundaries of organizations show “fuzziness.” In terms of consumer distance, enterprises are becoming increasingly closer. Consumers are increasingly paying attention to the ICSR of enterprises while also focusing on the ECSR. That is, the ICSR of enterprises has risen to a new height in the minds of consumers. The epidemic has had a great impact on the normal operation of SMEs, regarding the production or sales activities or the daily work of employees. SMEs need to respond actively, open resources, reduce expenditures, stabilize their cash flow, and ensure their survival in the epidemic. Under circumstances where other factors are established, SMEs should actively fulfill their ICSR and find new ways out of the predicament.

Second, paying attention to and implementing ICSR in an epidemic situation is conducive to establishing brand image and enhancing consumer brand attitudes. According to the findings of this study, under the impact of the epidemic, SMEs face difficulties. Therefore, within their ability, SMEs need to protect employee interests first and actively fulfill their ICSR to establish a good enterprise brand image. Such an image can help enterprises survive the epidemic and usher in greater development opportunities after the epidemic. For example, Laoxiang Chicken actively fulfilled its ICSR and has obtained loans worth 100 million yuan from banks to overcome its difficulties. Therefore, fulfilling ICSR not only helps SMEs during the epidemic crisis but also helps sustain the thrust of continuous development, empowering the growth of enterprises. The epidemic has played a positive role in promoting forced change, and under such pressure, SMEs should actively fulfill their ICSR, turning the threat into an opportunity.

Third, SMEs should promptly disclose their CSR activities to improve consumer brand attitudes. The development of we-media has gradually matured, and as a cheap and convenient public platform, we-media can transmit information to the outside promptly. SMEs should use Weibo, public accounts, and Douyin (short video-sharing platform) to actively disclose their CSR activities to improve their consumer brand attitudes. At the same time, SMEs should also pay attention to reasonable and effective publicity. According to the findings of this study, SMEs should publicize their ICSR and objectively display their difficulties. They should fulfill their ICSR and be aware of “good welfare” and “bad influence.” Only by letting consumers know the difficulties of the enterprise can they truly understand that the enterprise is sacrificing its interests to protect the interests of its employees, thus preventing public opinion in which people question that the enterprise blindly improves its internal welfare by neglecting its social responsibility, which is a show to deceive consumers.

Fourth, a crisis management mechanism should be constructed, including the establishment of a plan, provision for a reserve fund system, and an early warning mechanism. Once a crisis occurs, SMEs can prepare and react quickly, and enterprise losses can be reduced to the minimum.

### Research limitations and prospects

First, this study is based on cross-sectional data from questionnaire surveys. The relevant research results cannot be used to draw causal inferences concerning the research structure. If future research can use panel data from CSR annual reports issued after the epidemic, it can be more intuitive to compare the impact of enterprises’ ICSR on consumer brand attitudes at different points before and after the epidemic. Second, this study does not verify whether enterprises use we-media channels to disclose CSR is better than if they use traditional media. In the future, we can verify the impact of SMEs’ choice of different channels to disclose CSR on consumer trust and brand attitudes. Finally, as the epidemic has not yet ended, whether consumers’ favorable attitudes toward enterprise brands will translate into improved performance after the epidemic still needs further research in the future.

## Data Availability

The python data crawler was used to capture 14,123 comments on the micro blog of “just, chairman of the board of directors of the Laoxiang Chicken tore up the joint employee letter” released on February 8, 2020. The data are available from ml1991@ruc.edu.cn.
